# Reply to the letter from Bhagirath et al.: Imaging for cardiac resynchronisation therapy requires cardiac magnetic resonance

**DOI:** 10.1007/s12471-018-1196-z

**Published:** 2018-11-07

**Authors:** U. C. Nguyên, M. J. M. Cluitmans, F. W. Prinzen, C. Mihl, K. Vernooy

**Affiliations:** 1 Department of Physiology, Cardiovascular Research Institute Maastricht (CARIM), Maastricht University Medical Center (MUMC+), Maastricht, The Netherlands; 2 Department of Cardiology, Cardiovascular Research Institute Maastricht (CARIM), Maastricht University Medical Center (MUMC+), Maastricht, The Netherlands; 3 Department of Radiology & Nuclear Medicine, Cardiovascular Research Institute Maastricht (CARIM), Maastricht University Medical Center (MUMC+), Maastricht, The Netherlands; 4 Department of Cardiology, Radboud University Medical Center, Nijmegen, The Netherlands

We thank Dr Bhagirath and co-authors for their knowledgeable comments on our study [1]. We agree that cardiac magnetic resonance imaging (CMR) has many applications that are relevant to cardiac resynchronisation therapy (CRT) implantation guidance. Indeed, as pointed out by Bhagirath and co-authors, a study from the London group [2] proposed coronary venous anatomy imaging by CMR. The same group also demonstrated the comprehensive use of computed tomography in selecting the optimal coronary vein for left ventricular lead placement by targeting regions of late mechanical activation and avoiding scarring [3].

The scope of our study was not to prove the superiority of one technique to the other. Instead, our study focused on sharing our clinical experience with coronary venous anatomy visualisation by computed tomography and comparing this with fluoroscopic angiography, as the latter technique is traditionally used in coronary venous anatomy delineation.

Many cardiac imaging modalities have been studied for left ventricular lead guidance and CRT response prediction with each having its own strengths and limitations. The utility of each technique may depend on its purpose. Both CMR and computed tomography indeed have the potential to image the coronary veins. CMR has the advantage of avoiding ionising radiation and is more commonly used in clinical practice for focal scar evaluation using delayed enhancement and for functional analyses of the left ventricle using cine imaging. However, CMR has lower spatial resolution compared with computed tomography, which is a factor particularly relevant for the visualisation of small and delicate blood vessels [4].

Furthermore, a substantial number of CRT candidates already have existing pacing systems, and thus a contraindication for CMR. On the other hand, coronary venous computed tomography using contrast media may be contraindicated in patients with severe kidney impairment.

**Fig. 1:**
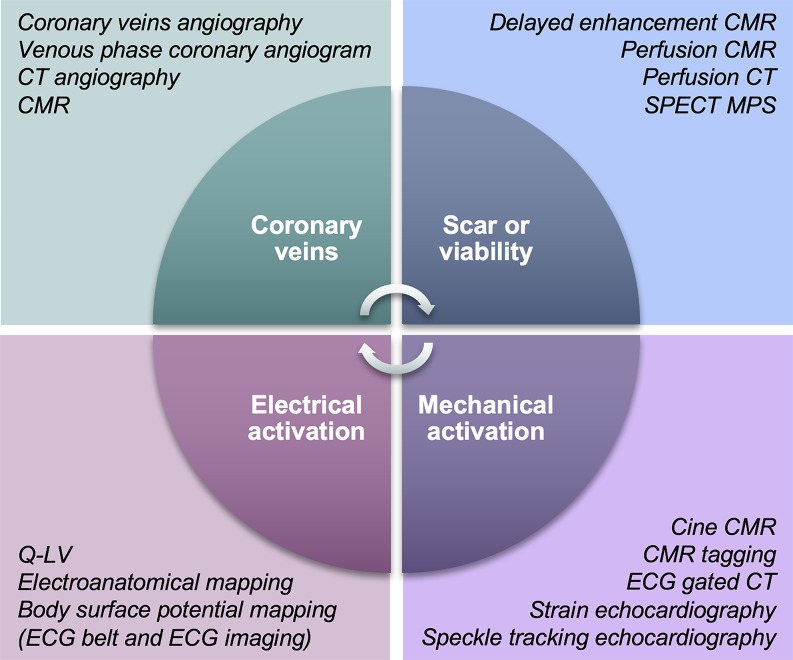
Schematic overview representing the key components relevant for left ventricular lead placement including the imaging techniques that may evaluate these aspects (*CMR* cardiac magnetic resonance imaging, *CT* computed tomography, *ECG* electrocardiography, *SPECT* single-photon emission tomography, *MPS* myocardial perfusion scintigraphy, *Q-LV* intrinsic local electrical delay at the left ventricular lead)

The future of image-guided CRT may not be a single imaging technique, but an integration of multiple modalities combining the strengths of multiple techniques. Key components in this process (and relevant for left ventricular lead guidance) include coronary venous anatomy, electrical or mechanical activation, and scar or viability delineation (Fig. 1). Each aspect may be assessed through different imaging techniques complementing each other to reach a common goal.
